# Supplementary material for: MSTO1 is a cytoplasmic pro‐mitochondrial fusion protein, whose mutation induces myopathy and ataxia in humans
Source: EMBO Mol Med. 2017 May 29;9(7):967–84. doi: 10.15252/emmm.201607058 (PMC5494519; doi:10.15252/emmm.201607058)
Supplement: Supplementary file 1 — Expanded View Figures PDF [file EMMM-9-967-s001.pdf]

Expanded View Figures

A

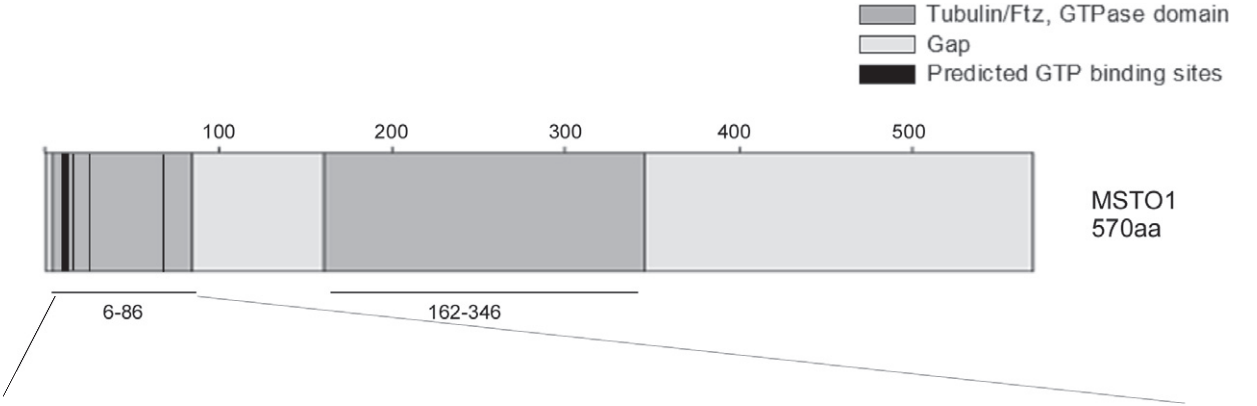


```
6 REVLTLQLGHFAGFVGAHWWNQQDAALGRATDSKEPPGELCPDVLYRTGRTLHGQETYTPRLILMDLKGSLSSLKEEGGL 86
```

B

```
HsMSTO1 192  LEAFGQGESVLKEPKYQEEL-----EDRLHFYVEECDYLQGFQILCD  233
HsMFN1  232  FILNNRWDASASEPEYMEDVRRQHMERCLHFLVEELKVVNALEAQNR  279
HsMFN2  254  FILNNRWDASASEPEYMEEVRRQHMERCTSFLVDELGVVDRSQAGDR  300
```

C

```
Homo sapiens              192  LEAFGQGESVLKEPKYQEELEDRLHFYVEECDYLQGFQILCDLHDGF  238
Pan troglodytes           192  LEAFGQGESVLKEPKYQEELEDRLHFYVEECDYLQGFQILCDLHDGF  238
Bos taurus                192  LEAFGQGESILKEPKYLEELEDRLHFYVEECDYLQGFQILCDLHDGF  238
Mus musculus              156  LEAFGQGESVLKEPRYLEELEDRLHFYVEECDYLQGFQLLCDLHDGF  202
Rattus norvegicus         175  LEAFGQGESVLKEPRYLEELEDRLHFYVEECDYLQGFQILCDLHDGF  221
Meleagris gallopavo       108  LEAFGQGEKLLQDVGCIEELEDRLHFYVEECDYLQGFQVLCDLHDGF  154
Ophiophagus Hannah        137  LEAFGQGEKLLQDGRYLEELEDRLHFY--------GFQLLCDLHNGF  175
Xenopus tropicalis        202  LEAYGQGEALLQEAAYGEELEDRLHFFTEECDYLQGFHLVCDLHNGF  248
Danio rerio               200  LEVFGQGEALL-QGQVLEDLEDRLHFFIEECDYLQGFQVLCDLTDGF  245
Drosophila melanogaster   189  LGTYSAGTEMWQEVSFNEEFCDRIRLYVEECDGLQGFHVLFDIDDGF  235
Saccharomyces cerevisiae  168  FDRYSIGYDEFTENYLQEFFDGNLHRELEKCDTLQGFNLVSDMESGW  214
```

Figure EV1. *In silico* analysis of the predicted GTPase domains of MSTO1.

A Predicted GTP binding and GTPase homology domains in MSTO1. The bold font indicates the possible GTPase binding sites.

B Alignment of MSTO1 with the GTPase domain of MFNs. The bold fonts and box indicates the higher similarities.

C Evolutionary conservation of the region in MSTO1, which shows similarity the GTPase regions of MFNs. The bold fonts and box indicates the higher similarities.

© 2017 The Authors

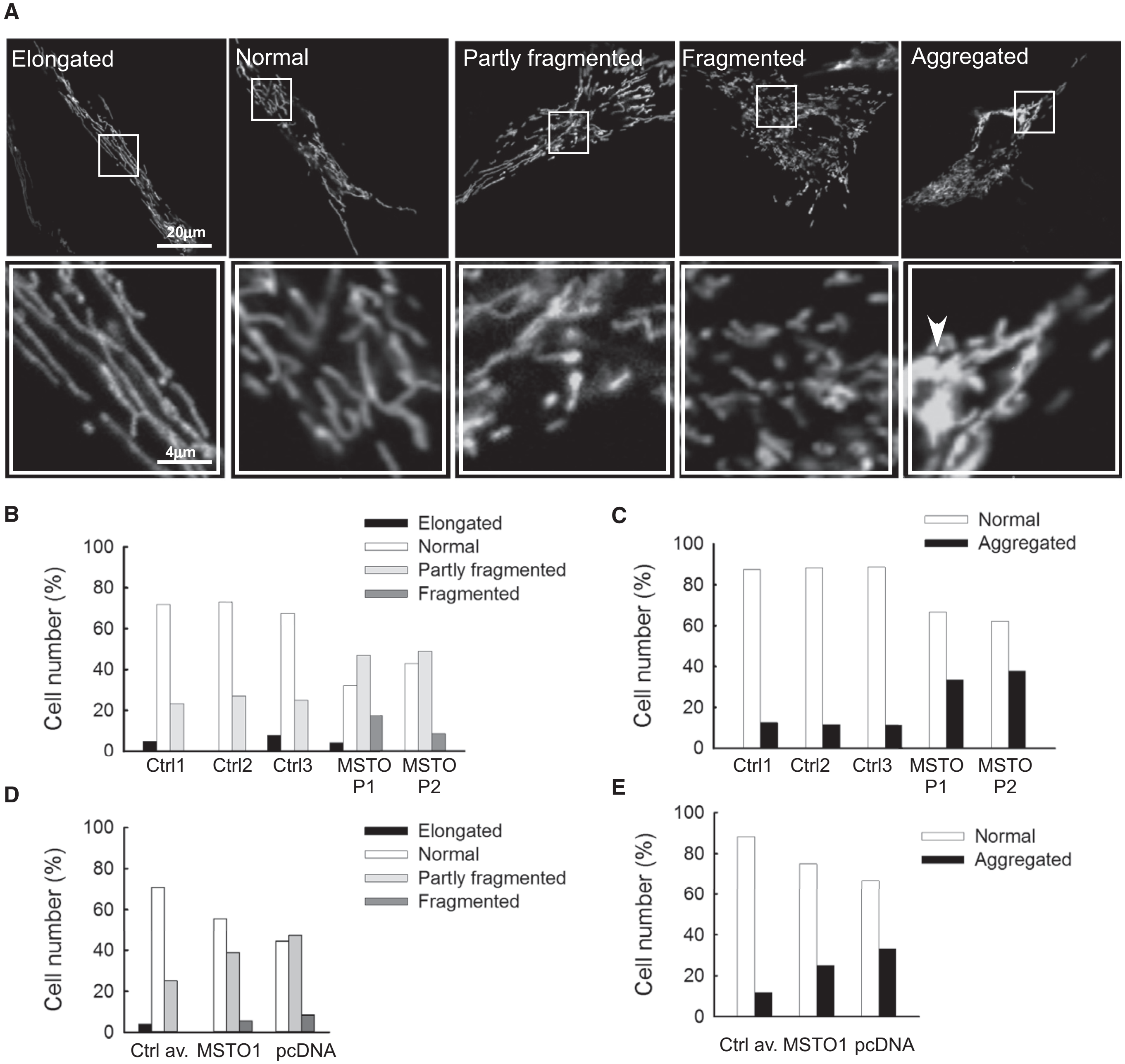

Figure EV2. Mitochondrial morphology in primary fibroblasts.

Mitochondrial morphology was scored as follows: fragmented, mainly small and round; partly fragmented, intermediate, mixture of round and shorter tubulated; normal, tubulated, long and higher interconnectivity; elongated, very long, tubulated. The percentage of cells with indicated mitochondrial morphologies was determined as a percentage of the total number of DsRed-transfected cells counted (≥ 20 cells per experiment) in $n = 5$ independent experiments.

A Representative figures of the different mitochondrial types. The arrowhead indicates the aggregated mitochondria. The white box showing higher magnifications.

B, C Distribution of the different type of mitochondria in primary fibroblasts.

D, E Distribution of the different type of mitochondria after MSTO1 silencing in HeLa cells (Scr.: scramble siRNA-silenced cells; MSTO1: MSTO1 siRNA-silenced cells).

 © 2017 The Authors

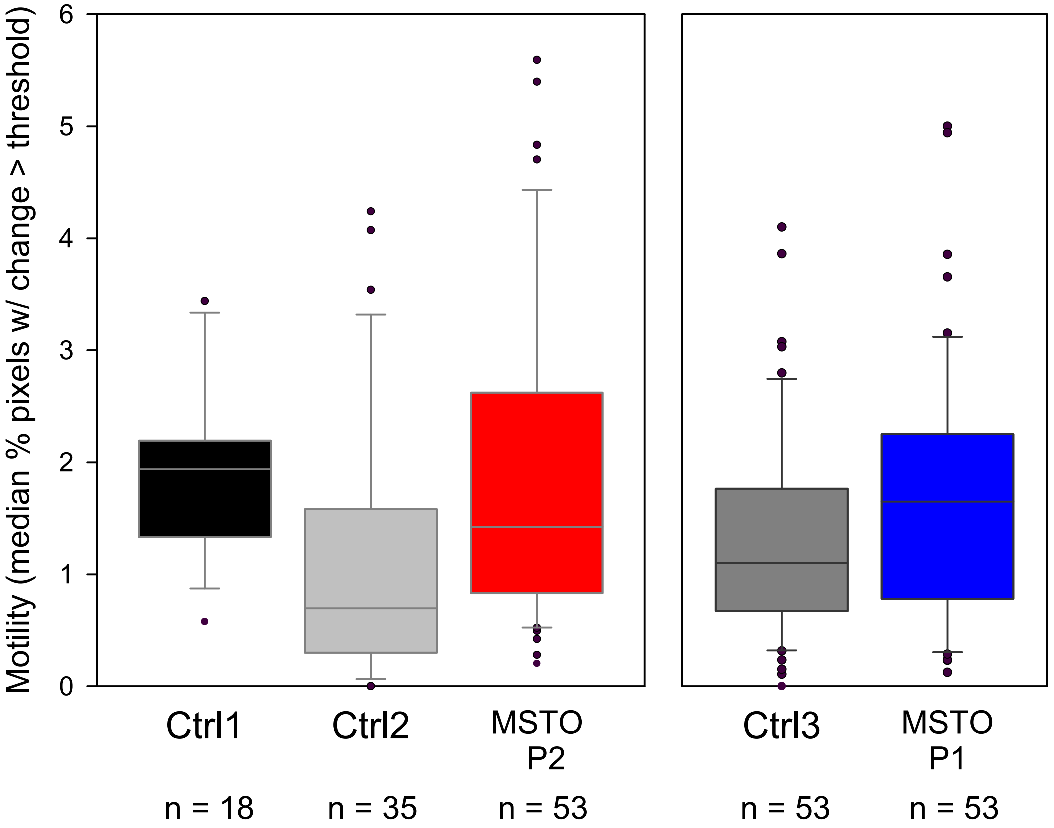

Figure EV3. Motility analysis from fusion records.

To estimate mitochondrial motility in MSTO1 patient and control fibroblasts from records used for fusion analysis, the green and red channels were combined by addition. From the resulting image (summed image), a difference image series was generated by subtracting successive images. Both the difference image series and the summed images were thresholded by a constant value (32 in an 8-bit image) and the number of pixels above the threshold in the period after the photoactivation was determined. The final measure is given as the median number of pixels above the threshold from the difference image series normalized by the median number of pixels above the threshold from the summed image series. Horizontal line shows the median value; box, the 25th and 75th percentiles; error bars, 10th and 90th percentiles; circles are individual outlying cells. The overall fluorescence intensity of the cells was also measured (mean intensity of pixels above threshold) in the summed image, and no significant differences in intensity between groups were found (P = 0.395, one-way ANOVA). Data are from six experiments: three comparing P1 and Ctrl3, two comparing P2 and Ctrl2, and one with P2 and Ctrl1; the number of cells in each group is indicated.
