# Supplementary material for: MSTO1 is a cytoplasmic pro‐mitochondrial fusion protein, whose mutation induces myopathy and ataxia in humans
Source: EMBO Mol Med. 2017 May 29;9(7):967–84. doi: 10.15252/emmm.201607058 (PMC5494519; doi:10.15252/emmm.201607058)
Supplement: Supplementary file 2 — Table EV1 [file EMMM-9-967-s002.docx]

**Table EV1**

|  | COL5A1 p.Glu98Lys | MSTO1 p.Val8Met | RELN p.Ile3374Val | RYR2 p.Cys501Gly |
| --- | --- | --- | --- | --- |
| I/2 (MSTO P1) | Heterozygous | **Heterozygous** | Heterozygous | Heterozygous |
| II/2 (MSTO P2) | Heterozygous | **Heterozygous** | Heterozygous | Heterozygous |
| II/3 | Wild-type | **Heterozygous** | Wild-type | Wild-type |
| II/4 | Heterozygous | **Heterozygous** | Wild-type | Heterozygous |

**Table EV1: Comparative results of the Sanger validation within the effective family members**
