# Supplementary material for: MSTO1 is a cytoplasmic pro‐mitochondrial fusion protein, whose mutation induces myopathy and ataxia in humans
Source: EMBO Mol Med. 2017 May 29;9(7):967–84. doi: 10.15252/emmm.201607058 (PMC5494519; doi:10.15252/emmm.201607058)
Supplement: Supplementary file 3 — Table EV2 [file EMMM-9-967-s003.doc]

**Table EV2**

**List of the primers**

mtDNA common Deletion Fw 5’-3’ taaaaatctttgaaatagggc

mtDNA common Deletion Rev 5’-3’ cggatacagttcactttagct

Tm: 58^o^C

MtDNA-1 Fw 5’-3’ CCCACACGTTCCCCTTAAAT

MtDNA-1 Rev 5’-3’ GTCTTTGGGGTTTGGTTGGT

Tm: 60^o^C

MtDNA-2 Fw 5’-3’ TTATCTTTTGGCGGTATGCAC

MtDNA-2 Rev 5’-3’ TCAGGTGAGTTTTAGCTTTATTGG

Tm: 60^o^C

MtDNA-3 Fw 5’-3’ CACGGGAAACAGCAGTGATT

MtDNA-3 Rev 5’-3’ TTTCATAAGGGCTATCGTAGTTTT

Tm: 60^o^C

MtDNA-4 Fw 5’-3’ ACCACCTCTTGCTCAGCCTA

MtDNA-4 Rev 5’-3’ TCCTTGCTATATTATGCTTGGTT

Tm: 60^o^C

MtDNA-5 Fw 5’-3’ TGACCGCTCTGAGCTAAACC

MtDNA-5 Rev 5’-3’ GGAGTTCAGTTATATGTTTGGGATTT

Tm: 60^o^C

MtDNA-6 Fw 5’-3’ AGAACCCTCTAAATCCCCTTG

MtDNA-6 Rev 5’-3’ AGAGACAGCTGAACCCTCGT

Tm: 60^o^C

MtDNA-7 Fw 5’-3’ GCCTGTTTACCAAAAACATCA

MtDNA-7 Rev 5’-3’ CCTGGATTACTCCGGTCTGA

Tm: 60^o^C

MtDNA-8 Fw 5’-3’ ACCAACGGAACAAGTTACCC

MtDNA-8 Rev 5’-3’ TAGATGTGGCGGGTTTTAGG

Tm: 60^o^C

MtDNA-9 Fw 5’-3’ TCCTCATTGTACCCATTCTAATC

MtDNA-9 Rev 5’-3’ CTAGTTCGGACTCCCCTTCG

Tm: 60^o^C

MtDNA-10 Fw 5’-3’ TGAAGTCACCCTAGCCATCA

MtDNA-10 Rev 5’-3’ GCACGGAGAATTTTGGATTC

Tm: 60^o^C

MtDNA-11 Fw 5’-3’ GATTCCGCTACGACCAACTC

MtDNA-11 Rev 5’-3’ GGGGCTATTCCTAGTTTTATTGC

Tm: 60^o^C

MtDNA-12 Fw 5’-3’ GCTTTTATTCCAGTTCTAACCAAAA

MtDNA-12 Rev 5’-3’ GAGAGGAGGGTGGATGGAAT

Tm: 60^o^C

MtDNA-13 Fw 5’-3’ GCATACTCCTCAATTACCCACA

MtDNA-13 Rev 5’-3’ GTGGGGTTTTGCAGTCCTTA

Tm: 60^o^C

MtDNA-14 Fw 5’-3’ CATTCCTCCCCACACTCATC

MtDNA-14 Rev 5’-3’ AGCTCGGCTCGAATAAGGAG

Tm: 60^o^C

MtDNA-15 Fw 5’-3’ TTTACAGTCCAATGCTTCACTCA

MtDNA-15 Rev 5’-3’ GAAGAGGGGCGTTTGGTATT

Tm: 60^o^C

MtDNA-16 Fw 5’-3’ TGAACAGTCTACCCTCCCTTAG

MtDNA-16 Rev 5’-3’ GGCGAGTCAGCTAAATACTTTGA

Tm: 60^o^C

MtDNA-17 Fw 5’-3’ CCATATTGTAACTTACTACTCCGGAAA

MtDNA-17 Rev 5’-3’ TGCTGTTAGAGAAATGAATGAGC

Tm: 60^o^C

MtDNA-18 Fw 5’-3’ AGACCAAACCTACGCCAAAA

MtDNA-18 Rev 5’-3’ GGGCATACAGGACTAGGAAGC

Tm: 60^o^C

MtDNA-19 Fw 5’-3’ GAAAAACCATTTCATAACTTTGTCA

MtDNA-19 Rev 5’-3’ GTTTAGACGTCCGGGAATTG

Tm: 60^o^C

MtDNA-20 Fw 5’-3’ CCCCCATTATTCCTAGAACCA

MtDNA-20 Rev 5’-3’ AAGCGAACAGATTTTCGTTCA

Tm: 60^o^C

MtDNA-21 Fw 5’-3’ TACTACCGTATGGCCCACCA

MtDNA-21 Rev 5’-3’ TGAGTAGGCTGATGGTTTCG

Tm: 60^o^C

MtDNA-22 Fw 5’-3’ CATTTACACCAACCACCCAAC

MtDNA-22 Rev 5’-3’ CGCCATCATTGGTATATGGTT

Tm: 60^o^C

MtDNA-23 Fw 5’-3’ CCTCTACCTGCACGACAACA

MtDNA-23 Rev 5’-3’ GGAAGCCTGTGGCTACAAAA

Tm: 60^o^C

MtDNA-24 Fw 5’-3’ TCAATCACCTGAGCTCACCA

MtDNA-24 Rev 5’-3’ ACTAAGAAGAATTTTATGGAGAAAGG

Tm: 60^o^C

MtDNA-25 Fw 5’-3’ AAAAAGAGTAATAAACTTCGCCTTAAT

MtDNA-25 Rev 5’-3’ GGCACAATATTGGCTAAGAGG

Tm: 60^o^C

MtDNA-26 Fw 5’-3’ TTTACCAAATGCCCCTCATT

MtDNA-26 Rev 5’-3’ TGGCTGTGAATGTTATAATTAAGGA

Tm: 60^o^C

MtDNA-27 Fw 5’-3’ CCAAATCAACAACAACCTATTTAGC

MtDNA-27 Rev 5’-3’ CCATAGCCGCCTAGTTTTAAG

Tm: 60^o^C

MtDNA-28 Fw 5’-3’ TCAAACTCCTGAGCCAACAA

MtDNA-28 Rev 5’-3’ CACAGAGAGTTCTCCCAGTAGG

Tm: 60^o^C

MtDNA-29 Fw 5’-3’ TTCTGCCTAGCAAACTCAAAC

MtDNA-29 Rev 5’-3’ CTTTTATTTGGAGTTGCACCA

Tm: 60^o^C

MtDNA-30 Fw 5’-3’ CCGGGTTTTCCTCTTGTAAA

MtDNA-30 Rev 5’-3’ TCTCAGCCGATGAACAGTTG

Tm: 60^o^C

MtDNA-31 Fw 5’-3’ AACCCAAACAACCCAGCTCT

MtDNA-31 Rev 5’-3’ TGGTGATAGCGCCTAAGCAT

Tm: 60^o^C

MtDNA-32 Fw 5’-3’ CAGGCAAATCAGCCCAATTA

MtDNA-32 Rev 5’-3’ CAGGGAGGTAGCGATGAGAG

Tm: 60^o^C

MtDNA-33 Fw 5’-3’ GGAGGACTACTCAAAACCATACC

MtDNA-33 Rev 5’-3’ GGTTAGGTCTAGGAGGAGTAGGG

Tm: 60^o^C

MtDNA-34 Fw 5’-3’ CCCTCGCTGTCACTTTCCTA

MtDNA-34 Rev 5’-3’ AGGAGTATCCTGAGGCATGG

Tm: 60^o^C

MtDNA-35 Fw 5’-3’ CCAATAGGATCCTCCCGAAT

MtDNA-35 Rev 5’-3’ TTCATCATGCGGAGATGTTG

Tm: 60^o^C

MtDNA-36 Fw 5’-3’ TCTCGCACGGACTACAACC

MtDNA-36 Rev 5’-3’ GTGTGAGGGTGGGACTGTCT

Tm: 60^o^C

MtDNA-37 Fw 5’-3’ CGGCATTATCCTCCTGCTT

MtDNA-37 Rev 5’-3’ TGCTTTGTTGTTTGGATATATGG

Tm: 60^o^C

MtDNA-38 Fw 5’-3’ CCCTAGCCAACCCCTTAAAC

MtDNA-38 Rev 5’-3’ TGGCTGGCAGTAATGTACGA

Tm: 60^o^C

MtDNA-39 Fw 5’-3’ CCTTTTTCCAAGGACAAATCA

MtDNA-39 Rev 5’-3’ TGACCCTGAAGTAGGAACCAG

Tm: 60^o^C

MtDNA-40 Fw 5’-3’ CCTCACCCACTAGGATACCAA

MtDNA-40 Rev 5’-3’ AGGATGAGGCAGGAATCAAA

Tm: 60^o^C

RELN Ex62 Fw 5’-3’ GGGTGACAGAGCAAGACTCC

RELN Ex62 Rev 5’-3’ TGTGGCATTGGTGCATTAAC

Tm: 60^o^C

RYR2 Ex16 Fw 5’-3’ GCATTTTTAGTCTGTAAGCAGAATG

RYR2 Ex16 Rev 5’-3’ TTACAGGCATGAGCCATCAC

Tm: 60^o^C

COL5A1 Ex3 Fw 5’-3’ GCTGGCTTGTTTGCAGAGAG

COL5A1 Ex3 Rev 5’-3’ GCTGCACGTCCTCTACTGTG

Tm: 60^o^C

MSTO1 Ex1-2 Fw 5’-3’ CAAGCCAATCGGCTAGGAG

MSTO1 Ex1-2 Rev 5’-3’ TATTCCCGAAATGGCTCTTG

Tm: 65^o^C

MSTO1 Ex3-4 Fw 5’-3’ GCGTCCCACAGCTAATGACT

MSTO1 Ex3-4 Rev 5’-3’ TGCTTCGTCCTCCTTGAGTT

Tm: 60^o^C

MSTO1 Ex5-7 Fw 5’-3’ CGACTCGGTGAACAGAAAGG

MSTO1 Ex5-7 Rev 5’-3’ CAGGTCACACAGGATCTGGA

Tm: 60^o^C

MSTO1 Ex8-9 Fw 5’-3’ TCCTAAAGGAACCCAAGTACC

MSTO1 Ex8-9 Rev 5’-3’ GGACAAGCATAGGACAGTGTGA

Tm: 65^o^C

MSTO1 Ex10-11 Fw 5’-3’ CAGCCTGGGGTCAAACATAC

MSTO1 Ex10-11 Rev 5’-3’ TATGTTCTCAGGCCATCAGC

Tm: 60^o^C

MSTO1 Ex12-13 Fw 5’-3’ CCCACCCCTTAAAAAGGAAA

MSTO1 Ex12-13 Rev 5’-3’ GAGGAAGAATGCTTTTCTTGG

Tm: 60^o^C

MSTO1 Ex14 Fw 5’-3’ TTCTTCCTCTGGAGTACTGGTG

MSTO1 Ex14 Rev 5’-3’ AAAAAGTGCATATGTTGGTGTAGC

Tm: 60^o^C

MSTO1 cDNS-1 Fw 5’-3’ CCAATCGGCTAGGAGCAG

MSTO1 cDNS-1 rev 5’-3’ TCCTCCAGCTCTTCCTGGTA

Tm: 60^o^C

MSTO1 cDNS-2 Fw 5’-3’ TGCAGAGGGAGTGCTGAGTA

MSTO1 cDNS-2 rev 5’-3’ AACTGCATCAGGGAATCAGG

Tm: 60^o^C

MSTO1 cDNS-3 Fw 5’-3’ TCAGCTTCCCTTACCTGCAT

MSTO1 cDNS-3 rev 5’-3’ CATGAAGACACTGGGCTCCT

Tm: 60^o^C

MSTO1 cDNS-4-Fw 5’-3’ GTGGAGAGCATCCCAGTGTT

MSTO1 cDNS-4 rev 5’-3’ AGCCCTCCTCCTTTCTTAGG

Tm: 60^o^C

Human serum albumin Ex12 Fw 5’-3’ AGCTATCCGTGGTCCTGAAC

Human serum albumin Ex12 Rev 5’-3’ TTCTCAGAAAGTGTGCATATATCTG

Tm: 60^O^C
