# Supplementary material for: MSTO1 is a cytoplasmic pro‐mitochondrial fusion protein, whose mutation induces myopathy and ataxia in humans
Source: EMBO Mol Med. 2017 May 29;9(7):967–84. doi: 10.15252/emmm.201607058 (PMC5494519; doi:10.15252/emmm.201607058)
Supplement: Supplementary file 4 — Table EV3 [file EMMM-9-967-s004.docx]

| **Antibody** | **Host** | **Immunogen** | **Used dilutions** | **Company** | **Cat#** |
| --- | --- | --- | --- | --- | --- |
| α Tubulin | Mouse | Bovine Alpha-Tubulin. | 1:500 | Thermo Fisher Scientific | A11126 |
| Cytochrome-c | Mouse | Synthetic Peptides of Pigeon Cytochrome C | 1:1000 | BD Bioscience | 556433 |
| Drp1 | Mouse | Human Drp1 aa. 19-201 | 1:500 | BD Bioscience | 611738 |
| Hexokinase II | goat | The C-terminus of HXK II of human origin | 1:500 | Santa Cruz Biotechnology | sc-6521 |
| HSP70 | Mouse | Human recombinant HSP70 over expressed in E. col | 1:1000 | Thermo Fisher Scientific | MA3-008 |
| Mfn1 | Rabbit |  | 1:1000 | (Richard Youle, NIH, Bethesda, MD |  |
| Mfn2 | Mouse | generated against Mfn2 [710–757]-GST and N-terminal peptide CNSIVTVKKNKRIIM-OH | 1:1000 | (Heidi McBride, McGill University, Montreal, Canada) |  |
| MSTO1 | Rabbit | Recombinant fragment corresponding to a region within amino acids 34 and 327 of MSTO1 | 1:500 | OriGene | TA308056 |
| OPA1 | Mouse | Human OPA1 aa. 708-830 | 1:500 | BD Bioscience | 612606 |
| Prohibitin | Rabbit | Synthetic peptide (the amino acid sequence is considered to be commercially sensitive) corresponding to Human Prohibitin (N terminal). | 1:1000 | abcam | ab75766 |

**Table EV3**

**List of the antibodies**
