# Supplementary figures and images for: MSTO1 is a cytoplasmic pro‐mitochondrial fusion protein, whose mutation induces myopathy and ataxia in humans
Source: EMBO Mol Med. 2017 May 29;9(7):967–84. doi: 10.15252/emmm.201607058 (PMC5494519; doi:10.15252/emmm.201607058)

**Figure 1F**

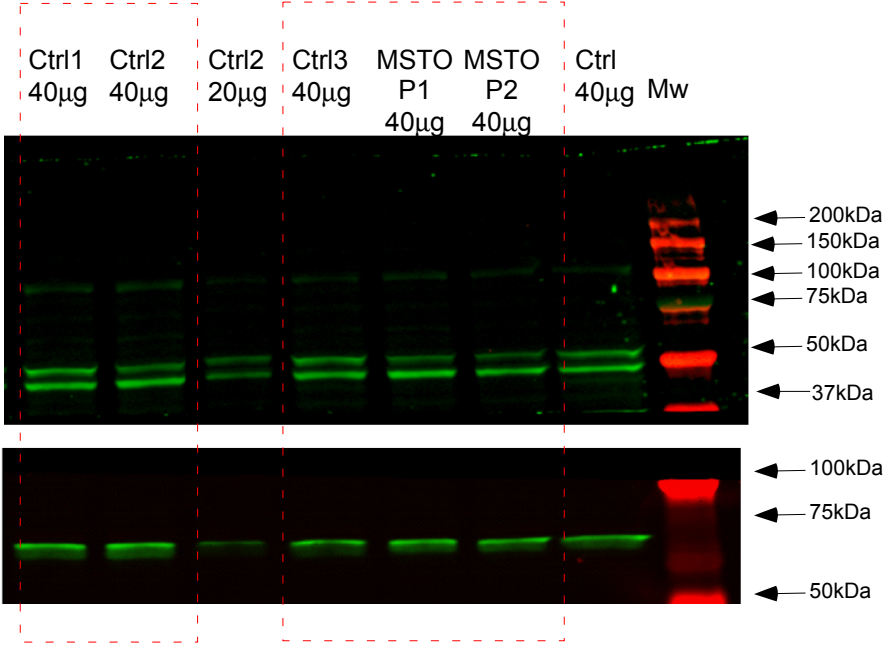

The main figure shows the lane(s) marked by red dash.

Supplement: Supplementary file 6 — Source Data for Figure 1 [file EMMM-9-967-s005.pdf]

Figure 3F

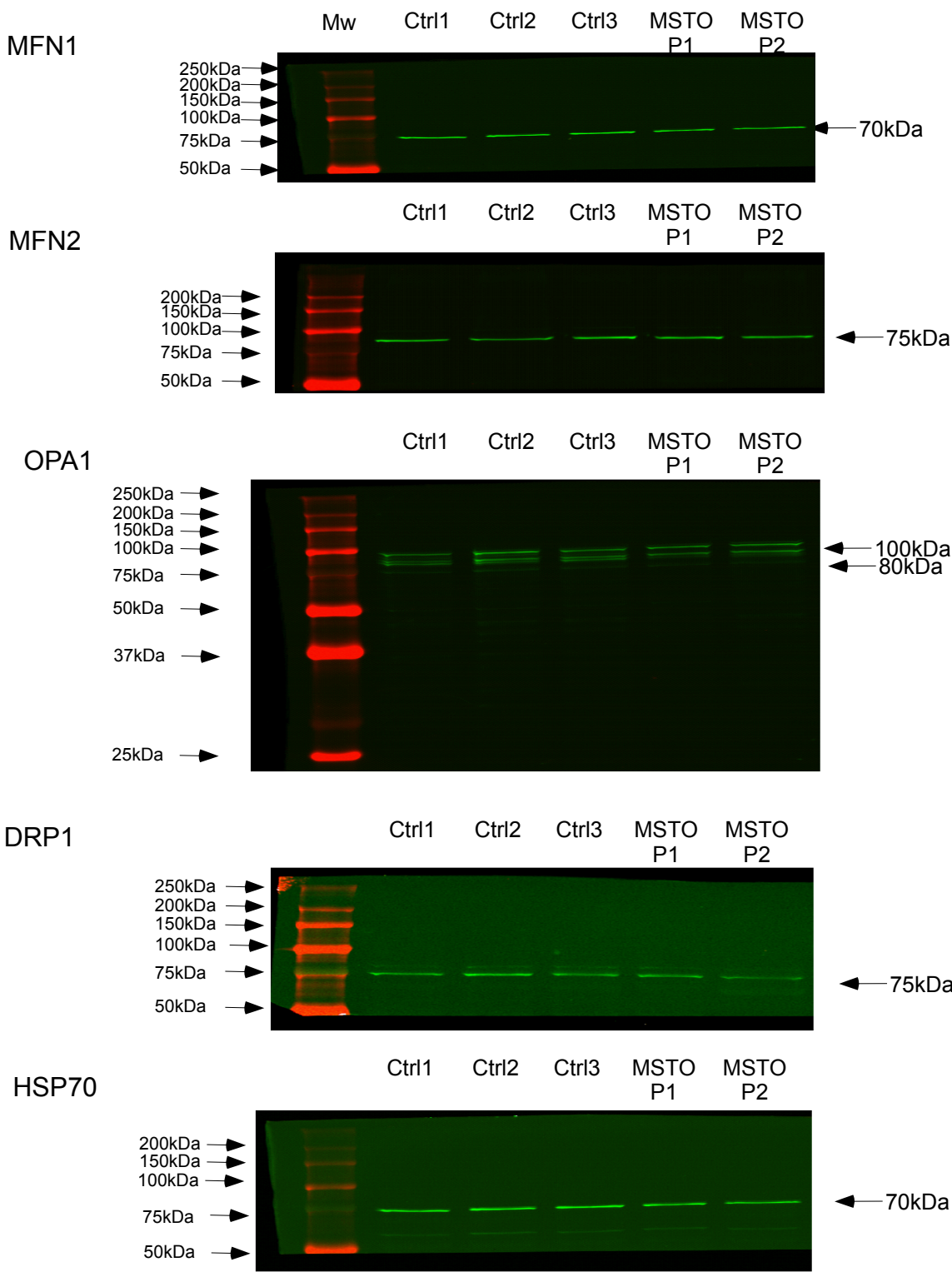

Supplement: Supplementary file 7 — Source Data for Figure 3 [file EMMM-9-967-s006.pdf]

Figure 4A

MSTO1

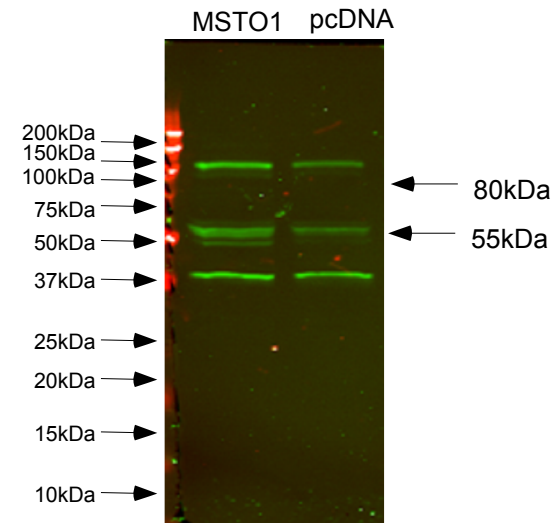

Supplement: Supplementary file 8 — Source Data for Figure 4 [file EMMM-9-967-s007.pdf]

**Figure 5A**

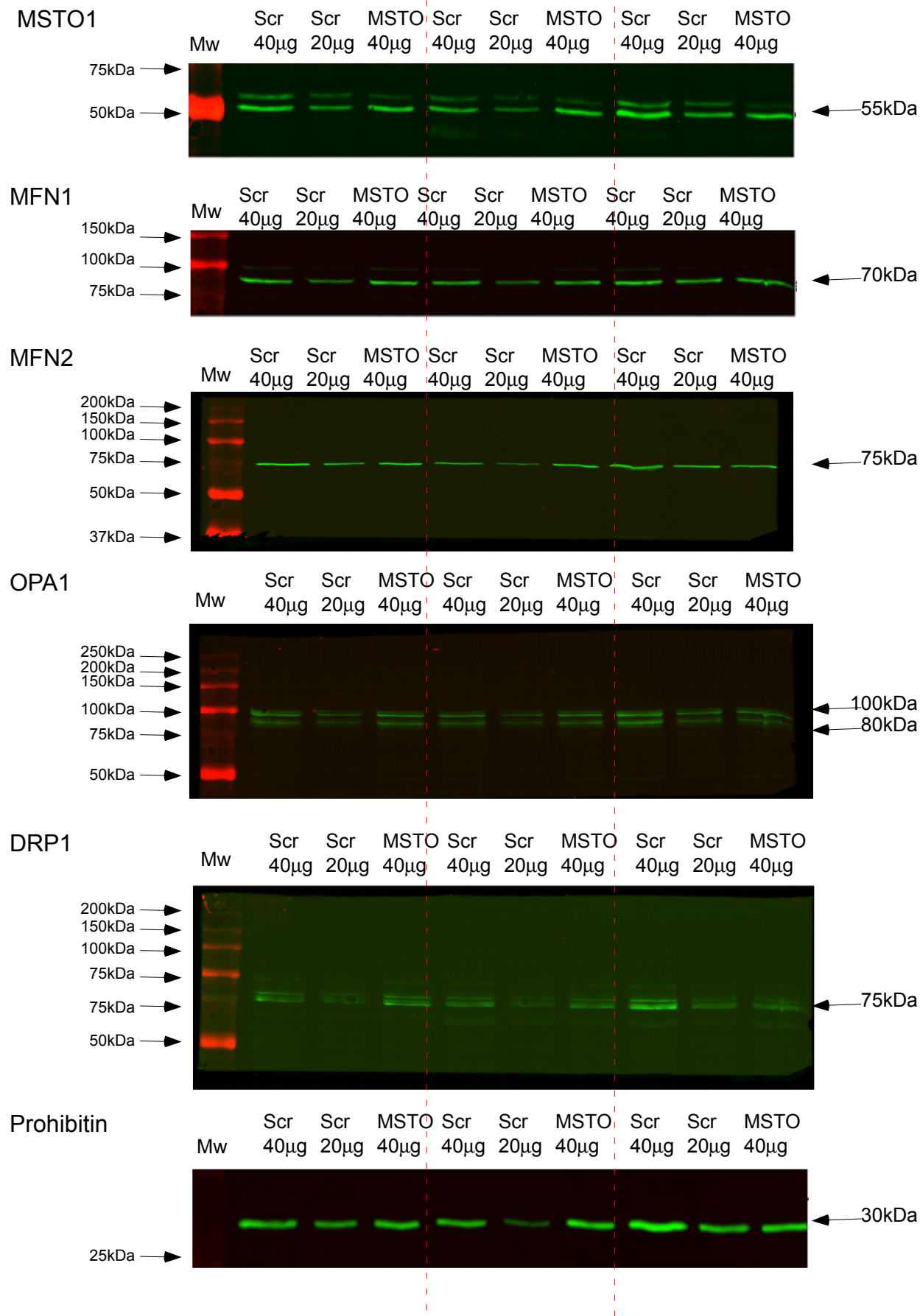

The main figure shows the lane(s) marked by red dash.

Supplement: Supplementary file 9 — Source Data for Figure 5 [file EMMM-9-967-s008.pdf]

Figure 7A

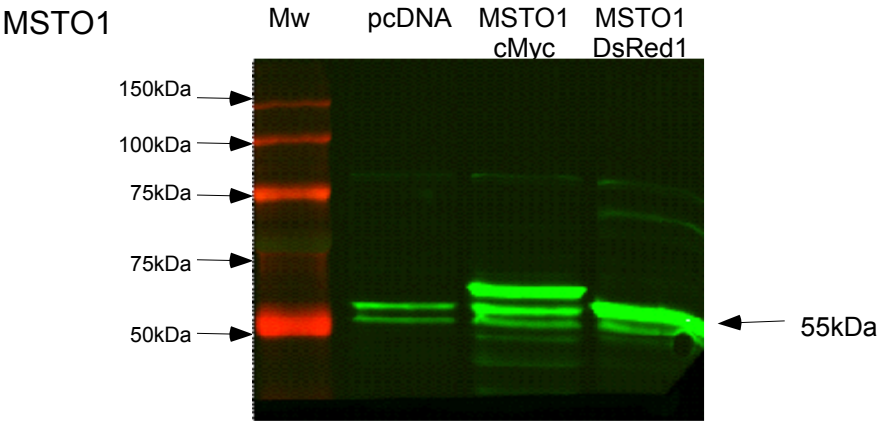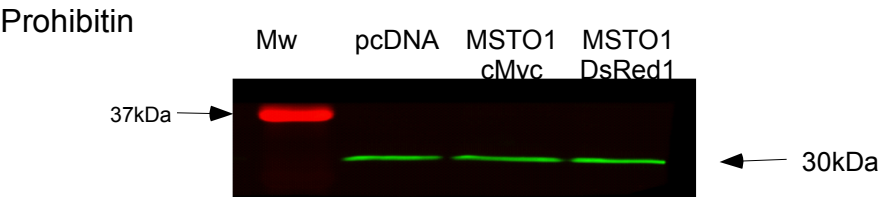

Figure 7F

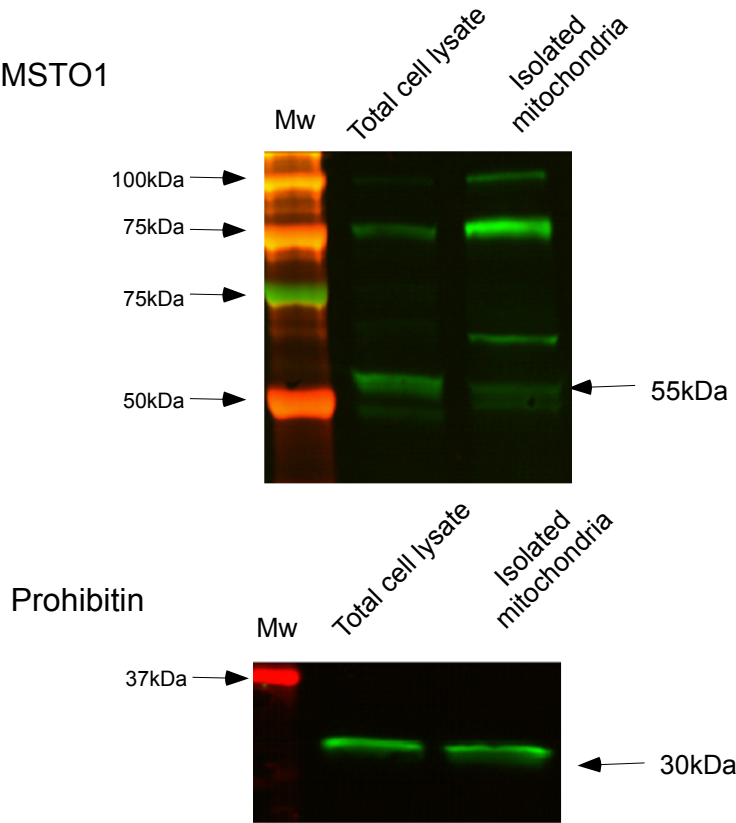

Supplement: Supplementary file 11 — Source Data for Figure 7 [file EMMM-9-967-s010.pdf]

Figure 8A

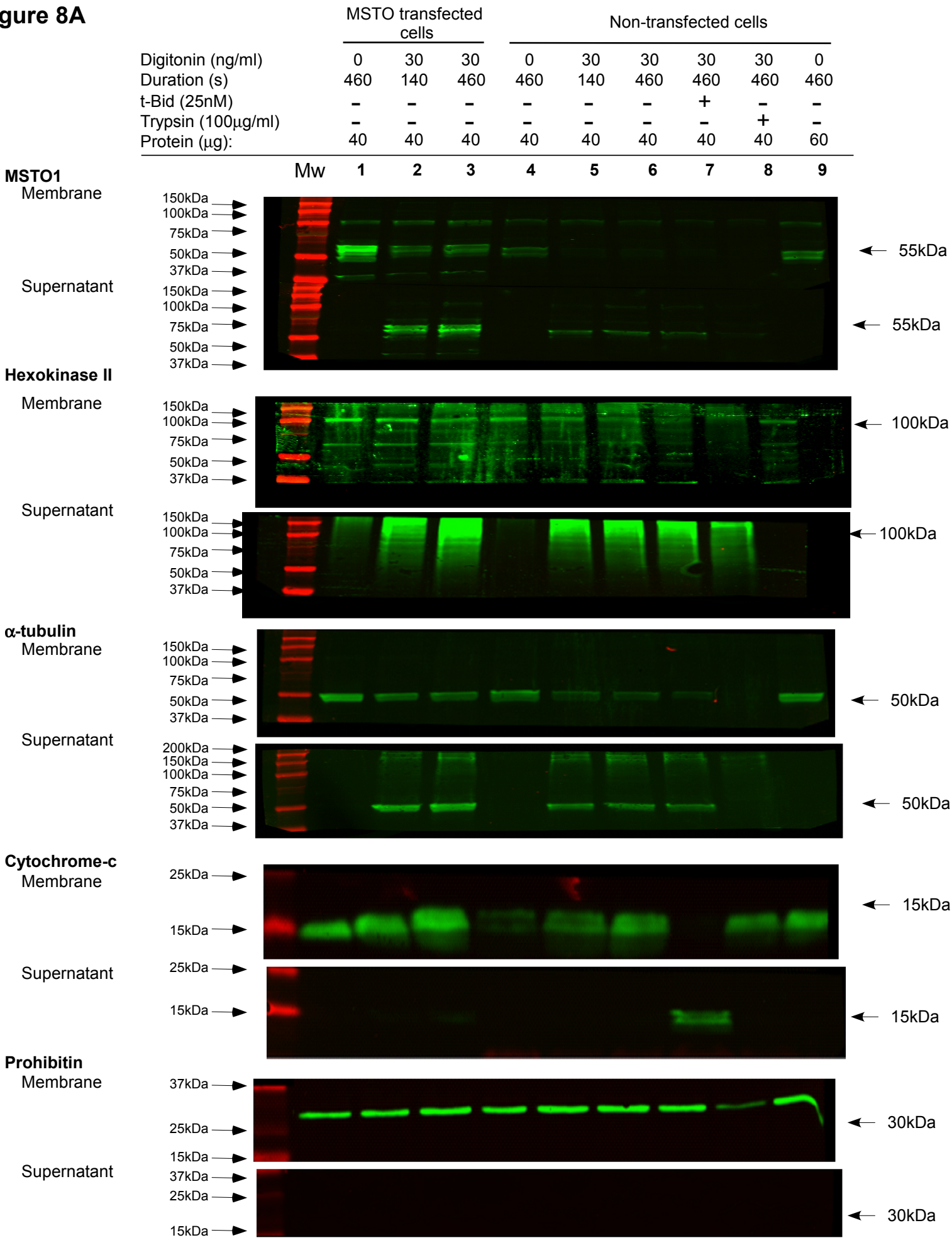

Supplement: Supplementary file 12 — Source Data for Figure 8 [file EMMM-9-967-s011.pdf]
