# Supplementary material for: MSTO1 is a cytoplasmic pro‐mitochondrial fusion protein, whose mutation induces myopathy and ataxia in humans
Source: EMBO Mol Med. 2017 May 29;9(7):967–84. doi: 10.15252/emmm.201607058 (PMC5494519; doi:10.15252/emmm.201607058)

Figure 6C - JO2 data

Ctrl	mean	sd	sem	t-test	9-Dec P9	9-Dec P10	9-Dec P11	12-Dec P9	12-Dec P11
basal	5.85	0.99	0.44	0.46	6.55	5.67	6.77	6.00	4.25
oligo	2.26	1.80	0.80	0.90	1.20	0.80	0.90	4.62	3.77
FCCP	8.87	1.31	0.59	0.01	8.55	8.50	9.30	10.78	7.20
kd					1	1	1	1	1

SH date					9-Dec	9-Dec	9-Dec	12-Dec	12-Dec
siMSTO					P9	P10	P11	P9	P11
basal	5.24	1.44	0.64		6.92	6.01	5.26	3.04	4.98
oligo	2.13	1.27	0.57		1.15	1.64	1.53	1.98	4.33
FCCP	6.14	1.37	0.61		7.87	6.89	6.43	4.54	4.99
kd	0.48	0.21	0.09		0.76	0.44	0.23	0.35	0.6

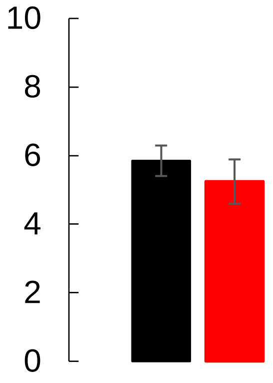

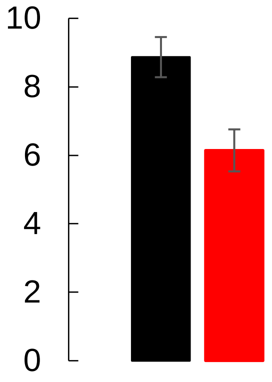

Supplement: Supplementary file 10 — Source Data for Figure 6 [file EMMM-9-967-s009.pdf]
